# A research protocol for developing a Point-Of-Care Key Evidence Tool ‘POCKET’: a checklist for multidimensional evidence reporting on point-of-care in vitro diagnostics

**DOI:** 10.1136/bmjopen-2015-007840

**Published:** 2015-07-10

**Authors:** Jeremy R Huddy, Melody Ni, Stella Mavroveli, James Barlow, Doris-Ann Williams, George B Hanna

**Affiliations:** 1 Department of Surgery and Cancer, Imperial College, London, UK; 2 Imperial College Business School, South Kensington Campus, London, UK; 3 British In Vitro Diagnostics Association (BIVDA), London, UK

**Keywords:** QUALITATIVE RESEARCH

## Abstract

**Introduction:**

Point-of-care in vitro diagnostics (POC-IVD) are increasingly becoming widespread as an acceptable means of providing rapid diagnostic results to facilitate decision-making in many clinical pathways. Evidence in utility, usability and cost-effectiveness is currently provided in a fragmented and detached manner that is fraught with methodological challenges given the disruptive nature these tests have on the clinical pathway. The Point-of-care Key Evidence Tool (POCKET) checklist aims to provide an integrated evidence-based framework that incorporates all required evidence to guide the evaluation of POC-IVD to meet the needs of policy and decisionmakers in the National Health Service (NHS).

**Methods and analysis:**

A multimethod approach will be applied in order to develop the POCKET. A thorough literature review has formed the basis of a robust Delphi process and validation study. Semistructured interviews are being undertaken with POC-IVD stakeholders, including industry, regulators, commissioners, clinicians and patients to understand what evidence is required to facilitate decision-making. Emergent themes will be translated into a series of statements to form a survey questionnaire that aims to reach a consensus in each stakeholder group to what needs to be included in the tool. Results will be presented to a workshop to discuss the statements brought forward and the optimal format for the tool. Once assembled, the tool will be field-tested through case studies to ensure validity and usability and inform refinement, if required. The final version will be published online with a call for comments. Limitations include unpredictable sample representation, development of compromise position rather than consensus, and absence of blinding in validation exercise.

**Ethics and dissemination:**

The Imperial College Joint Research Compliance Office and the Imperial College Hospitals NHS Trust R&D department have approved the protocol. The checklist tool will be disseminated through a PhD thesis, a website, peer-reviewed publication, academic conferences and formal presentations.

**Strengths and limitations of this study**

- Multimethod approach incorporating all stakeholders.
- Agreement of independent National Institute of Health Research Diagnostic Evidence Co-operatives to prospectively field-test and provide feedback on the developed tool.
- Collaborative approach of academia and industry.
- Delphi methodology may lead to a compromise position rather than true consensus.
- Small sample size with unpredictable representation.

## Introduction

Point-of-care in vitro diagnostics (POC-IVD) provide rapid results near patient or at the bedside to facilitate real-time clinical decision-making. POC-IVD devices are well established in medical, industrial and military environments. Clinical examples include blood glucose, anticoagulation and pregnancy testing. These offer potential advantages such as decreasing the time to definitive treatment, improved cost-effectiveness,^1^ ^2^ better patient adherence with treatment and increased patient satisfaction.^3^ However, barriers to the implementation of POC-IVD technology exist, including the increased cost on a test-by-test basis, reduced accuracy, unclear clinical pathway benefits and associated maintenance and governance responsibilities.^4^ Many of these barriers may be overcome if better evidence was available. With a POC-IVD diagnostic industry that is expanding rapidly, with an estimated 29% share of the in vitro diagnostic market^5^ and predicted to be worth US$24 billion by 2018,^6^ it is important that we have the methodology to evaluate these devices in a valid, efficient and timely manner.

Point-of-care tests are a disruptive innovation that is used by both healthcare professionals and patients at clinical facilities or at home to prompt an immediate clinical decision. Evidence generation on such technology should include not only the assessment of the device itself, but also the test processes (sample collection, test procedure and result output) and system dynamics (training, data transfer, patient safety and intervention). Clinical pathway examination is fundamental to incorporate healthcare benefits, consequences of misdiagnosis and the impact on alternative diagnostic workforce. Accumulating appropriate evidence results in a long lead-time from innovation to clinical adoption, with some technologies never making the transition to the bedside.

Quality evidence on POC-IVD is required for regulation, policy making and implementation. Currently, the pathway for evidence generation in diagnostics is fragmented and does not follow the linear sequence of evaluations that has become mainstream for drug evaluation.^7^ Diagnostic accuracy is often established with poor methodological quality^8^ and alone rarely provides sufficient justification for device adoption. Evidence is required to demonstrate the behaviour changes induced by the test result and the consequences to patient outcome. Usability testing and economic analysis are commonly performed in isolation. This multifaceted approach is time consuming, expensive and stands as a barrier to devices translating from industry to the healthcare environment. Given the considerable overlap in these methodology work streams, a multidimensional concurrent approach to evidence generation would be able to add significant efficiencies to the evidence generation pathway.

There are several reports in the medical literature to improve the quality of evidence in diagnostic devices. Examples include the standards for reporting of diagnostic accuracy studies (STARD)^9^ initiative to improve accuracy and completeness in diagnostic accuracy studies; a quality assessment tool for diagnostic accuracy studies (QUADAS-2)^10^ to evaluate the risk of bias in primary diagnostic accuracy studies; and the consolidated health economic evaluation reporting standards statement (CHEERS)^11^ for reporting health economic evaluations. This is paralleled in the design literature by standards published by the International Organisation for Standardisation (ISO) and British Standards (BS), including BS EN 62366,^12^ that relates to usability engineering in medical devices. The National Institute for Health and Clinical Excellence (NICE) has published a Diagnostics Assessment Programme Manual^13^ outlining what evidence is required for their appraisal process and how this should be synthesised. However, all these standards and guidelines were specific to a particular focus and there is an absence of an integrated framework to present the multidimensional evidence ‘package’ that is required by decisionmakers when evaluating new medical technologies.

The aim of this study is to develop a *P*oint-*O*f-*C*are *K*ey *E*vidence *T*ool (POCKET); as a ‘one-stop’ multidimensional evaluation checklist and reporting standard incorporating validity, utility usability, cost-effectiveness and patient experience in POC-IVD at a device, process and system level. The motivation for POCKET is to aid industry and academic researchers when undergoing the development and evaluation of POC-IVD to ensure the combined evidence requirements of users and policy decisionmakers are met efficiently, reducing the lead time for the adoption of new technologies and allowing demonstrable benefits to patients and the National Health Service to be effectively realised.

## Methods

**Figure 1:**
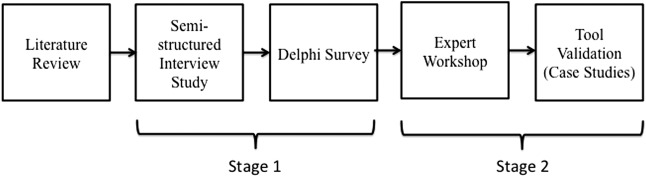
Study outline.

The POCKET checklist will be developed and refined by a multimethod approach utilising stakeholders in the POC-IVD field and experts in methodology. The study design is outlined in figure 1. A semistructured interview study will identify emergent themes to guide a Delphi study that will generate an expert consensus on tool inclusion. This will be presented at a workshop to develop the initial draft tool that will be refined and validated by a series of case studies to field-test the tool.

### Study status

Recruitment and semistructured interviews (stage 1) started in August 2014. The questionnaire round of the Delphi survey will be sent out in August 2015 and the expert workshop is planned for the final quarter of 2015.

A comprehensive literature review preceded the interview protocol development to identify the potential evidence requirements for tool inclusion and at what stage of the device development pathway these reports can be generated. Electronic searches of medical and design databases were searched using a search strategy consisting of keywords and MeSH headings designed to identify articles related to POC-IVD.

### Stage 1: Delphi process

#### Delphi round 1: Semistructured interviews

A semistructured interview study is being undertaken with stakeholders relating to POC-IVD in order to collect primary data. Five stakeholder groups are being invited: patients and patient group representatives, clinicians, industry, regulators and commissioners. A minimum of eight participants from each group will be interviewed as a pragmatic sample size, given time and resources. However, the final number of participants will be guided by the interview results, as more interviews will be carried out until no new themes emerge. The majority of participants will be from the UK, but where relevant experience or expertise exists abroad such participants will be included in the study. Every effort will be made to recruit a representative and diverse sample across each participant group. Inclusion criteria and method of recruitment will vary by stakeholder group:

- *Patients* are recruited from the public through the patient information forum, patient associations and diagnostic research public engagement events. They will have experience of home POC-IVD use for the monitoring of chronic disease. They will not be directly recruited from the NHS. Patient records will not be accessed for recruitment.
- *Industry representatives* include personnel currently employed by a point-of-care industry and are recruited by an invitation included in their trade association BIVDA (British In Vitro Diagnostics Association) newsletter.
- *Clinicians* are recruited directly by electronic mail based on expertise in point-of-care diagnostics as demonstrated by academic output or their role on point-of-care committees.
- *Commissioners* include current members of clinical commissioning groups in northwest London and are recruited directly by electronic mail following an introduction from the Department of Primary Care and Public Health at Imperial College.
- *Regulators* will include current members of the NICE Diagnostic Advisory Committee and the Medicines and Healthcare Products Regulatory Agency (MHRA), and are recruited through their partnerships with the Diagnostic Evidence Co-operative.

Exclusion criteria include those unable to give informed consent to participate in the study, inability to understand and communicate in English, and members of the public from vulnerable groups (those under 18, prisoners, those in dependent relationships, the mentally ill).

The interview structure has been designed by a clinician (JRH) in conjunction with an expert in qualitative research (SM) to permit the collection of quantitative and qualitative data while allowing for flexibility and original suggestions. The interviews aim to understand the role different stakeholders have and the evidence that is required at each step in the adoption pathway of POC-IVD. This pathway includes device design, evaluation and implementation. Interviews are tailored to the role and level of experience of the interviewee, with significant tailoring required for interviews with patients. This ensures the interviews remain relevant to the different stakeholders. In the situation that the interviewee has a role in more than one stakeholder group, the interview focuses on their primary interest with subsequent roles recorded for analysis.

The interview questions were piloted to fine-tune the protocol prior to the study interviews. Interviews will be carried out face-to-face by one interviewer (JRH), with telephone or ‘Skype’ interviews reserved for when this is not feasible. Interviews will last for approximately 30–40 min, be digitally recorded and then transcribed verbatim for analysis. The main researcher and an additional team member with expertise in qualitative research will coanalyse the data independently in order to minimise bias. Initially, interview data will be coded based on the predetermined interview themes; it is expected that additional themes will emerge and be coded during the interview analyses. If required, participants will be contacted to clarify interpretation of data. Data will be analysed with NVivo V.10.1.1 software (QSR International, Melbourne, Victoria, Australia).

#### Delphi round 2: Delphi questionnaire summarising opinions and reaching consensus

Round 2 will allow experts to respond to questions that aim to reach a consensus on the required evaluation process for point-of-care diagnostic tests and add to the evidence base for the development of the POCKET checklist for multidimensional evidence generation.

All emergent themes relating to evidence requirements from the interview study will be translated into a series of statements and the survey questionnaire will be sent to an expert panel comprised of those interviewed in round 1 (with the exception of patients) as well as experts in research methodology identified by academic output. The Delphi study cohort will include a minimum of eight participants from each stakeholder group. As for round 1, this is a pragmatic sample size given the time and resources available for the study. The questionnaire statements will be responded to on a five-point Likert scale (1 (strongly disagree) to 5 (strongly agree)) in respect to checklist inclusion. Responders will also be able to add free text comments with each statement if needed to justify responses or suggest items that should be added and have been dismissed. The survey will be administered online using Qualtrics software (Qualtrics Labs Inc, Provo, UT) with personalised invitations sent out by electronic mail. Reminders will be sent at 4 and 6 weeks after initial contact for non-responders. Survey responses will be entered into SPSS and descriptive analyses carried out (frequencies, median scores, range of scores).

#### Delphi round 3: Reaching consensus

The survey will be repeated following feedback to the experts on the collated responses from the previous round. Any new items suggested in free text responses to round 2 will be added at this stage. This method will allow participants to reconsider their responses in light of the group results and they will be encouraged to justify their responses when there is a significant degree of divergence. This process will be repeated until consensus is reached. The aim of the POCKET tool is to meet the evidence requirements of all stakeholders. Therefore, stakeholder groups will be analysed individually and any statement deemed necessary by at least one group will be included in the tool. Consensus will be set at ≥80% across items that receive ≥4 (agree or strongly agree). Cronbach's α will be used to assess reliability. Previous studies^14–16^ have set a Cronbach's α of ≥0.8 as representative of an acceptable measure of internal reliability and this, therefore, will be the *priori* definition of consensus for this study.

### Stage 2: Expert workshop

#### Development of POCKET

A 2-day consensus workshop will be arranged for invited experts from researchers, editors, methodologists, industry and professional organisations. The workshop will follow the approach taken by Bossuyt *et al* in the development of the STARD guidelines.^8^ The workshop will start with a presentation of the results from the Delphi questionnaire followed by a mixture of small group sessions and plenary sessions. These sessions will aim to evaluate and justify the list of statements brought forward from the Delphi survey, and discuss the optimal format and phrasing of the checklist tool. Small multistakeholder groups will focus on a group of related statements with their outputs discussed at plenary sessions. A first draft of the POCKET checklist will be assembled by the end of day 1.

#### Validation of POCKET

The draft of the POCKET checklist will be discussed on the second day of the workshop and additional changes made. The checklist will be field-tested through a series of case studies of POC-IVD devices to ensure consistency, validity and usability. POC-IVD devices that have had demonstrable market success or failure in respect to clinical adoption will be identified through industry and expert feedback obtained during the Delphi process. Small groups will be provided with published research articles, conference abstracts and input from industry relating to devices and asked to retrospectively assess their adherence to the POCKET checklist. The groups will be not be informed to market results; however, it is possible that through their own experience they will already have an insight into the level of success many devices have had.

The output data from the groups will be analysed and a comparative evaluation undertaken to determine if devices that met the checklist requirements performed better in the IVD market and had successful implementation into the NHS. This exercise will inform further amendments to the tool if required. The final POCKET checklist will be published on the NIHR Diagnostic Evidence Co-operative London website with a call for comments. Following dissemination of the POCKET checklist users, including the NIHR Diagnostic Evidence Co-operatives in Leeds, Newcastle and Oxford, will be asked to provide feedback and further validation will be undertaken by real-world use of the tool in prospective POC device evaluations.

### Ethical concerns and dissemination

Participation in this study is voluntary. During the recruitment phase, all potential participants will be provided with a detailed information sheet in order to allow them to make an informed decision regarding their participation. The right of the participant to refuse to participate or withdraw at any time without giving reasons will be respected. Initially, informed consent was obtained for the interview study in isolation and these participants will require a further consent process for inclusion in subsequent Delphi rounds. However, an amendment was approved on 27 October 2014 requiring consent for the interview study and Delphi process together. No financial or other remuneration will be directly offered to participants. However, in some circumstances an honorarium will be offered to the practice of participants to compensate for their missed clinical time. This was agreed following advice from the Imperial College School of Primary Care. The protocol has been approved by the Joint Research Compliance Office Imperial College, London, and the Imperial College Hospitals NHS Trust R&D department (ICREC References 14IC2186, 14SM2190). The checklist tool will be disseminated through a PhD thesis (JRH), the NIHR-DEC website, peer-reviewed publications, academic conferences and formal presentations to industry, policymakers and practitioners.

## Limitations

this study design has several limitations. Many of these are common to qualitative research techniques and particularly, the Delphi process. The Delphi process may lead to a compromise position rather than a true consensus. The sample size of at least eight participants in each stakeholder group is small and the extent to which included participants will be representative is unknown. The recruitment protocol aims to recruit a broad selection of participants but the authors acknowledge that a degree of convenience sampling may be required. This could only be overcome by a random sampling approach that would not be feasible in the present study. The validation exercise that will be undertaken as part of the workshop is not blinded; therefore, the previous experiences of participants in respect to a particular technology may introduce a bias. A period of refinement has been included at the end of the study so that any evidence requirements not identified during the study can be added if required.
